# Impact of Spontaneous Haploid Genome Doubling in Maize Breeding

**DOI:** 10.3390/plants9030369

**Published:** 2020-03-17

**Authors:** Nicholas A. Boerman, Ursula K. Frei, Thomas Lübberstedt

**Affiliations:** Department of Agronomy, Iowa State University, Ames, IA 50011-1051, USA; ufrei@iastate.edu (U.K.F.); thomasl@iastate.edu (T.L.)

**Keywords:** doubled haploid, haploid male fertility, spontaneous haploid genome doubling, maize, chemical doubling agent, genome doubling, colchicine

## Abstract

Doubled haploid (DH) technology has changed the maize-breeding landscape in recent years. Traditionally, DH production requires the use of chemical doubling agents to induce haploid genome doubling and, subsequently, male fertility. These chemicals can be harmful to humans and the plants themselves, and typically result in a doubling rate of 10%–30%. Spontaneous genome doubling and male fertility of maize haploids, without using chemical doubling agents, have been observed to a limited extent, for nearly 70 years. Rates of spontaneous haploid genome doubling (SHGD) have ranged from less than 5% to greater than 50%. Recently, there has been increased interest to forgo chemical treatment and instead utilize this natural method of doubling. Genetic-mapping studies comprising worldwide germplasm have been conducted. Of particular interest has been the detection of large-effect quantitative trait loci (QTL) affecting SHGD. Having a single large-effect QTL with an additive nature provides flexibility for the method of introgression, such as marker-assisted backcrossing, marker-assisted gene pyramiding, and systematic design. Moreover, it allows implementation of new methodologies, such as haploid-inducer mediated genome editing (HI-edit) and promotion of alleles by genome editing. We believe the use of SHGD can further enhance the impact of DH technology in maize.

## 1. Overview of Doubled Haploid Technology in Maize

### 1.1. History and Uses of DH Technology

The objective of this manuscript is to first discuss the history, usage, and benefits of doubled haploids (DHs) in maize breeding. The standard colchicine-based system for DH line production will be discussed. Later in the manuscript, we present an alternative approach that leverages use of spontaneous haploid genome doubling (SHGD) present within certain maize lines as a replacement for colchicine. What is currently known regarding the biology and genetics of SHGD is presented, followed by methods for introgression and how we foresee SHGD to impact maize breeding.

Maize haploids were first reported by Randolph [1], and the use of colchicine to artificially double the genome of haploid *Datura* plants was summarized by Blakeslee and Avery [2]. However, the practicality of DHs in plant breeding was not reported until Chase [3] demonstrated their use in maize breeding. Implementation of DH technology has since revolutionized maize breeding by decreasing the time to develop homozygous lines and increasing the efficiency of a molecular breeding program [4].

The use of DH lines increases genetic gain, as illustrated by the following equation:

(1) $$G_{c}=\frac{ih^{2}\sigma_{p}}{t}$$

where *i* is the selection differential, *h*^2^ is the narrow sense heritability of the trait(s) being selected, σ_p_ is the phenotypic standard deviation, and *t* is the time needed per breeding cycle. The genetic variance of a DH population is larger compared to a population of segregating F_n_ families derived from the same cross [5], making it more likely to select superior progeny that outperform the parents during the line development process [6]. Moreover, homozygosity and homogeneity of DH lines increases heritability, compared to segregating family-type populations. The genetic variance of DHs is composed of only additive variance due to homozygosity at all loci, and the covariance between relatives is twice that of non-DH individuals [7,8,9]. The latter is important for the increase in genetic gain because (i) having the genetic variance be composed of only the additive component increases the response to selection, thus positively influencing heritability, and (ii) the increased covariance indicates the variance on which selection occurs is doubled in all associated parameters of the response to selection in DH lines, when compared to similar parameters in segregating lines [7]. In addition to the changes in genetic variance, complete homozygosity of DH lines allows greater repeatability, which can reduce environmental variation through increased replication [7,9]. Therefore, the response to selection during line development can be enhanced, and the time required can be reduced by using a DH breeding scheme versus the traditional approach of making selections within segregating populations.

Line development in maize has traditionally consisted of pedigree-based selection within segregating F_2_ populations, utilizing self-pollination and visual selection for several generations [10]. Recurrent selection complements pedigree-based selection by improving the mean of the breeding population through recombining superior progeny following selection. Inbred lines are then derived from continuously improved populations through offspring selection [10,11]. To increase efficiency and reduce the time required for each cycle, self-pollination steps can be replaced by the use of DHs, where either an F_1_, F_2_, or a randomly mating population is crossed to a haploid-inducer line (HIL) to derive DH lines [11]. 

Another application of DHs is in a marker-assisted backcrossing (MABC) program, using either phenotypic or marker-assisted approaches, by replacing the self-pollination steps at the end of the program, to reduce the time for fixation of alleles [12,13]. If DHs are used, fewer individuals are needed to identify an individual possessing the trait of interest, making them particularly useful if the trait has recessive gene action [13]. With the advent of high-throughput marker systems, major commercial breeding programs today combine DH technology with genomic selection (GS) to maximize their genetic gain [14]. 

DHs can be very useful in GS if a large number of quantitative trait loci (QTL) control a trait with low heritability [14,15,16]. Because of low genome doubling rates, DH lines are typically established before they are genotyped. To further accelerate the GS process, Wu et al. [17] suggested conducting GS at the haploid stage of DH line production, where individual haploid plants would be genotyped and only predicted superior haploids would be self-pollinated to produce DH lines. However, Wu et al. [17] determined that a minimum threshold for genome doubling of 20% must be exceeded to make such an endeavor economically feasible. Using the traditional genome doubling agent, colchicine, the doubling rate typically ranges from 10% to 30% [18,19]. Recently, five inbred lines exhibiting haploid male fertility (HMF) greater than 65% without the use of colchicine were identified by their pollen-shedding ability and proportion of anther emergence [20]. Furthermore, a single large-effect QTL has been identified on chromosome 5 in the inbred that had the highest level of HMF, at 94% [21,22]. Interestingly, this QTL is different from that identified on chromosome 6 within an inbred developed in China that also possesses a high degree of HMF [23]. Haploid male fertility is the most critical component of SHGD because haploid female fertility tends to be greater than 90% among the majority of maize lines [3,24,25]. Ultimately, SHGD can be thought of as the interaction between male and female fertility to produce successful self-pollinations of haploid plants in the D_0_ generation without the use of a chemical doubling agent, since both male and female gametes must be viable to produce viable offspring. A best standard practice for measuring SHGD should focus its consideration on pollen production and frequency of successful pollinations, because these factors combined are indicative of male and female gamete production and viability. Pollen production can vary greatly due to environment and weather conditions on a given day; therefore, it could be given a dichotomous presence or absence score. Frequency of successful pollinations would be the percentage of pollinations that were successful relative to the total attempted pollinations of a given genotype.

### 1.2. Methods for Producing DHs in Maize

**Figure 1:**
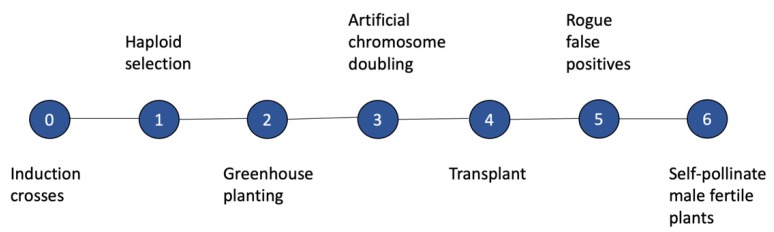
General outline of a pipeline for producing doubled haploids in maize by using a chemical doubling agent for genome doubling.

A common protocol for DH line development is as follows: (i) produce an F_1_ from two parental lines; (ii) cross the F_1_ to an HIL and sort putative haploid seed by a phenotypic marker; (iii) plant putative haploid seed in the greenhouse and inject seedlings with colchicine, or treat with some other chemical that prevents mitotic spindle function; (iv) transplant the putative haploid plants into the field; (v) rogue false-positives; and (vi) self-pollinate those haploids that are male fertile [26] (Figure 1). Variations of this protocol exist, such as dipping roots in colchicine instead of injection.

A major bottleneck in DH production is the selection of putative haploids. A phenotypic marker commonly used for visual kernel selection is *R_1_-nj*, which confers red pigmentation of the aleurone and embryo, indicating a hybrid between inducer and donor line [27]. Pigmentation of the aleurone and no pigmentation of the embryo indicate a putative haploid kernel. Another marker for visual selection is *Pl1*, conferring red pigmentation in the roots of seedlings. When using *Pl1*, a hybrid between inducer and donor line would have red roots and a haploid would have white roots. We further surmise that, depending on the donor background, the pigmentation of the coleoptile and/or the shoot might serve as an additional indicator for haploid/hybrid discrimination. However, little work has been done thus far to evaluate this marker, so further studies are needed prior to arriving at a definitive conclusion.

A more recently established phenotypic marker is oil content of the kernel, where the HIL has a higher oil content than the maternal donor [28]. Single-kernel nuclear magnetic resonance (NMR) spectroscopy was first demonstrated for use in selecting haploid kernels that have a lower oil content than the inducer by donor hybrids [28]. The authors found that the HIL oil content should be at least 2 percent higher than the donor genotype [28]. Near infrared spectroscopy (NIR) has also been used to select single haploid kernels based on composition and pigmentation and would also likely allow selection based on oil content [29]. While haploid selection is often cumbersome, the greatest bottleneck in DH production is chromosome doubling.

A commonly used chemical for chromosome doubling is colchicine, which is a chemical that is dangerous for both the environment and the user; it also has many governmental restrictions for usage and disposal [28]. Colchicine prevents the formation of microtubules by inhibiting microtubule polymerization during mitosis, arresting the cell in the M phase of cell division, resulting in duplicated chromosomes [30]. Some commercial herbicides also interfere with microtubule function, such as amiprophos-methyl (APM), flufenacet, oryzalin, propham, pronamid, and trifluralin [28,30]. A combination of APM and pronamid has been found to achieve an overall success rate of DH production close to colchicine and appears most promising as a colchicine alternative [28]. Another positive of using APM and pronamid is that they are much less toxic to humans than colchicine, and APM does not bind to animal tubulin [28,31]. Nitrous oxide (NO_2_) gas can also be used for chromosome doubling [32], although this method is more difficult to scale for a large breeding program than applying chemicals. Alternatively, a pipeline can be developed that utilizes SHGD, thus replacing the use of a chemical doubling agent and permitting direct seeding of putative haploids into the field.

## 2. Biology and Genetic Architecture of Spontaneous Haploid Genome Doubling

### 2.1. Biology of Spontaneous Haploid Genome Doubling

Spontaneous haploid genome doubling has been reported in maize at low levels for nearly as long as haploids have been studied [3,19,20,24,33,34,35]. Many genotypes from germplasm pools originating worldwide display SHGD, typically at levels < 5%. However, some genotypes have fertility levels > 50% [3,20,24,33,34,35]. 

The exact physiological timepoint of when SHGD is occurring appears to vary and is unknown in most cases. In one study, flow cytometric analysis of the flag leaf revealed variation in the number of diploid and haploid cells within some putative haploid plants [36]. Several of these plants even appeared morphologically to be completely diploid [36]. These results indicate that doubling is occurring early during the vegetative growth stage, at least for the genotypes studied. Furthermore, simple sequence repeat markers were used to confirm that alleles of various polymorphic markers were segregating in an approximately 1:1 ratio within these spontaneously doubled haploids, confirming that the plants were DHs derived by SHGD during an early vegetative growth stage [36]. While these diploid-like phenotypes have been reported at various levels in global germplasm pools, little is known about the underlying genetic mechanism. Furthermore, a chemically induced mutation of the *FIRST DIVISION RESTITUTION 1* (Zm*FDR1*) gene has resulted in first division restitution during meiosis within the male inflorescence [37]. This indicates that it is biologically possible to also have late-stage SHGD occurring as a mechanism within some of the lines reported to possess SHGD.

### 2.2. Genetic Architecture and Candidate Genes of Spontaneous Haploid Genome Doubling

Molenaar et al. [19] demonstrated that it is possible to utilize recurrent selection to increase the rate of SHGD. An increase ranging from 5% to 50% for SHGD was observed, depending on the genetic background in which SHGD was introgressed, suggesting that there were epistatic interactions between SHGD which were increasing alleles present in non-SHGD germplasm [19]. Therefore, it is possible to achieve rates of SHGD exceeding artificial genome doubling rates mediated by colchicine injection, which can range from 10% to 30%, depending on the genetic background [19]. 

**Figure 2:**
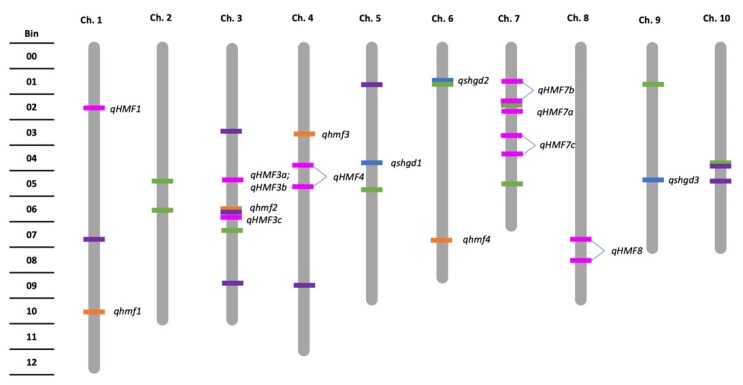
Genetic map showing location of quantitative trait loci (QTL) contributing to spontaneous haploid genome doubling (SHGD) from Ren et al. [23] (orange, lowercase *hmf*), Ren et al. and Trampe et al. [21,22] (blue), Yang et al. [38] (pink, uppercase *HMF*) and SNPs contributing to SHGD from Chaikam et al. [34] (green), and from Ma et al. [33] (purple). Map length depicted is from Ren et al. [23], totaling 1484.5 cM.

Ren et al. [23] were the first to report a mapping study for SHGD, where they mapped a large-effect QTL (*qhmf4*) based on segregation distortion to a region on chromosome 6 in a bi-parental population derived from Chinese germplasm (Figure 2). Moreover, in Chinese germplasm, Yang et al. [38] mapped nine QTL in total (Figure 2), one of which was located in bin 3.05 on chromosome 3 near *qhmf2* described by Ren et al. [23], and three QTL in bins 7.01 and 7.02 flanked a significant SNP on chromosome 7 (Figure 2) that was identified by Chaikam et al. [34]. Using a similar approach to [23], Ren et al. [21] mapped a large-effect QTL (*qshgd1*) to chromosome 5 in US-derived germplasm, and a small-effect QTL (*qshgd2*) to chromosome 6, which mapped to a different location than *qhmf4* (Figure 2). 

Furthermore, linkage mapping within a bi-parental mapping population using the same high SHGD parent as was used by Ren et al. [21] identified a large-effect QTL possessing additive gene action in the same region as *qshgd1* (Figure 2) [22]. Similarly, two small-effect QTL in the same regions as *qshgd2* and *qshgd3* identified by Ren et al. [21] were also detected (Figure 2) [22]. These differing QTL between the Chinese and US germplasms indicate that there are likely multiple mechanisms underlying similar phenotypes, possibly depending on the origin of germplasm.

A genome-wide association study (GWAS) was conducted, evaluating diverse germplasm from China and the US [33]. The diversity panel was crossed to both the inbred lines Mo17 and Zheng58, and their haploid progeny were evaluated for SHGD. Zheng58 was a parent contributing low SHGD in the bi-parental mapping populations used in the study by Ren et al. [23]. Significant differences were observed between the Mo17 and Zheng58 backgrounds, with Mo17 progeny exhibiting a higher average percentage of SHGD (23.8%) than Zheng58 progeny’s percentage of 13.5% [33]. Fifteen lines were identified as exhibiting high SHGD, and among those, 14 single nucleotide polymorphisms (SNPs) were identified as significantly contributing to HMF (Figure 2). One of these SNPs may correspond to *qhmf2* identified in the study by Ren et al. [23], as well as *qHMF3c* identified by Yang et al. [38] on chromosome 3 (Figure 2).

In another GWAS evaluating inbred lines adapted to the tropics and subtropics for SHGD, fertility ranged from 0.61% to 77.60% [34]. In total, eight SNPs were significantly associated with SHGD, and two were consistent with findings from previous studies [34]. Three of these SNPs were located on chromosome 3, and one of them was located in the same bin as *qhmf2* and *qHMF3c* (Figure 2). Meanwhile, a second SNP was located on chromosome 10, within the same bin as an SNP identified by Ma et al. [33,34] (Figure 2). 

Possible candidate genes for large-effect SHGD QTL have been suggested [21,23]. The gene *absence of first division* (*afd1*) is characterized by having an absence of first division during meiosis (first division restitution), resulting in a single equational division, and lies within the QTL *qhmf4* [23]. A gene encoding formin-like protein 5 is located within the QTL *qshgd1* and was downregulated in the SHGD donor line, when compared to a non-SHGD line [21]. Formins interact with actin, which is an important component for microtubule arrangement, as well as placement of the division plane during cytokinesis [39]. Thus, at least two mechanisms appear to be responsible for the different large-effect QTL controlling SHGD. Similarly, in both GWAS studies, several significant SNPs were associated with genes that have proposed functions related to meiosis, microtubule organization, and cell division [32,33]. Proper microtubule and division plate formation and organization are critical for proper meiosis function. While it appears quite possible that those mechanisms control SHGD, all candidate genes suggested so far need to be functionally evaluated, and their putative impact on SHGD needs to be confirmed. 

Due to the genetic diversity of SHGD donors in the abovementioned mapping studies, and the quantitative nature of most alleles, introgression of SHGD into a breeding program could be quite challenging. At least four QTL on chromosomes 3, 5, 6, and 10 have been identified by QTL mapping studies [21,22,23,38] and may be useful to increase SHGD ability in elite germplasm. The single large-effect locus on chromosome 5 identified by Ren et al. and Trampe et al. [21,22] should simplify introgression into elite germplasm by established MABC procedures. 

## 3. Introgression of Spontaneous Haploid Genome Doubling

**Figure 3:**
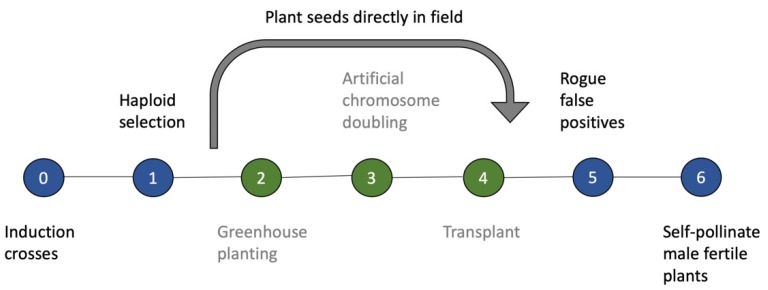
Pipeline for doubled haploid production, using SHGD as an alternative to artificial genome doubling agents. Green shading indicates stages of a traditional doubled haploid (DH) pipeline that are replaced by direct seeding into the field when utilizing SHGD.

By introgressing and utilizing SHGD, it would be possible to directly plant haploid seeds in the field, thus eliminating the need for a greenhouse and the use of dangerous chemicals for chromosome doubling (Figure 3). Introgression of SHGD into a breeding pool is necessary given the few lines that have high fertility rates. This issue could be addressed by introgressing SHGD into key hub lines within each heterotic pool through backcrossing to mitigate a potential genetic bottleneck. Multiple approaches for introgression can be taken, depending on the desired level of recurrent parent genome (RPG) to be recovered and number of genes or QTL controlling the chosen mechanism of SHGD.

**Figure 4:**
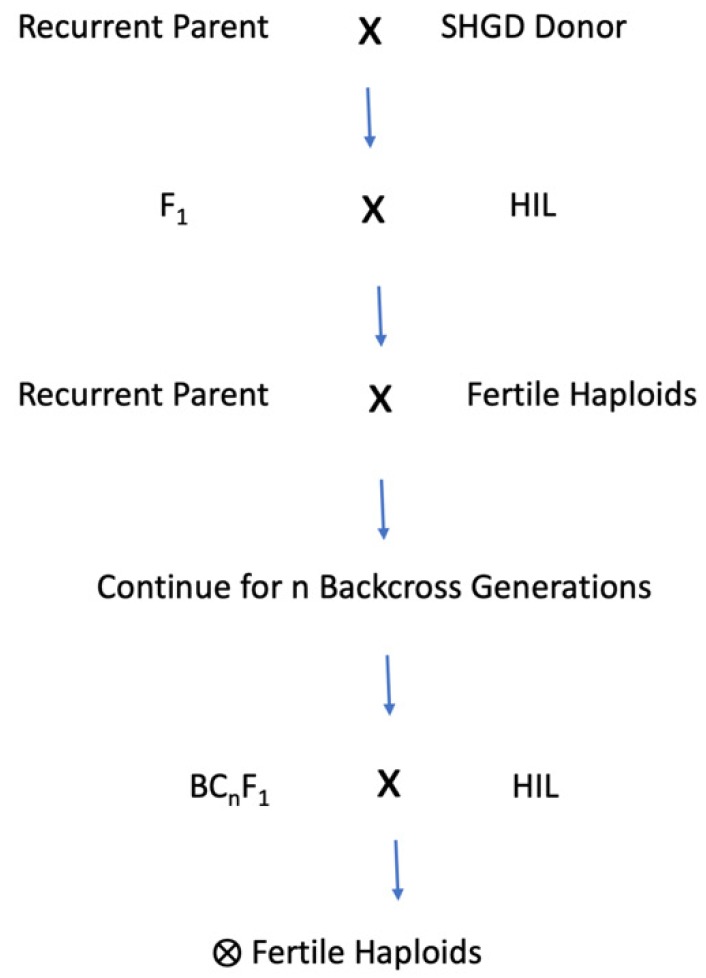
Schematic for phenotypic backcross introgression of SHGD into non-SHGD germplasm, resulting in DH lines possessing SHGD.

Inheritance of SHGD is mediated both by single large-effect QTL [21,22,23] and multiple small-effect QTL [19,33,34,38]. The methods used to introgress SHGD into elite germplasm depend on the SHGD source and its underlying genetic mechanism. To introgress a single large-effect SHGD QTL by using phenotypic backcrossing, one would cross an elite recurrent parent with a donor line carrying the major SHGD QTL, create an F_1_, and cross the F1 to a HIL. The resulting putative haploid seed would be planted into the field, without colchicine treatment. Male fertile haploids would be used as pollen parents and crossed to the recurrent parent (Figure 4). BC_1_ plants would be crossed to the HIL, and this scheme would be repeated for the desired number of backcross generations. Following the final backcross generation, the BC_n_ plants would be induced, and the resulting haploids exhibiting SHGD would be self-pollinated, to produce BC_n_ DH lines (Figure 4). 

Utilization of molecular markers would increase the efficiency of introgression of a major SHGD QTL. With abundant mapped molecular markers available in major crop species, such as maize [14], established MABC procedures can be used for introgression of the SHGD QTL, without the need to produce haploids to confirm the presence of this QTL by phenotypic evaluation, except at the end of the BC procedure.

Different multiple-stage approaches for introgression of a single gene were outlined by Frisch et al. [40] whereby selection for the gene or QTL of interest (foreground selection) is followed by selection for the recurrent parent marker alleles across all chromosomes (background selection). Background selection maximizes the desired proportion of RPG recovered. Two-stage and three-stage selection [40], with population sizes reduced after each generation, seem most appropriate. The number of markers used for background selection is affected by the percentage of RPG recovered [40]. 

Using two-stage selection from BC_1_ on, foreground selection is conducted to identify the individuals that possess the target gene, followed by background selection to identify individuals with the highest proportion of RPG. This process will be followed for as few generations as are needed until the desired percent of RPG is recovered. 

Three-stage selection differs from two-stage during the background selection step, where a step is added that identifies recombinants at markers flanking the gene of interest. Ideally, the markers are within 1–10 cM proximal of the target gene when making the second backcross generation, followed by selection of a flanking marker approximately 1–10 cM distal during the third backcross generation. Thus, by selecting recombinants of the carrier chromosome, the percent RPG recovered is higher than in two-stage selection, and the number of markers required for background selection is greatly reduced as compared to two-stage selection [40]. This three-stage approach is more economically efficient than two-stage selection because of the reduction in markers used. 

Marker-assisted backcrossing can be extended to two genes, as outlined by Frisch et al. [41], with the most efficient approach likely being Plan 6 [41]. There, each of the two QTL are introduced into the recipient germplasm separately, using one of the abovementioned selection schemes, until the BC_3_F_1_ generation. Plants from each of the BC_3_F_1_ populations are then crossed, merging both QTL in the recipient background [40].

The following discusses introgression of more than two SHGD QTL into elite germplasm. As the number of QTL needing to be incorporated increases, it becomes more difficult to restore the elite background. Thus, identifying major SHGD QTL is crucial for practical use, to minimize the number of SHGD QTL that needs to be introgressed.

With high-throughput marker technology available, systematic design for trait introgression increases the cost efficiency of introgression programs [42]. To implement systematic design, individuals would first be selected based on SHGD-associated markers (20–30 can be easily handled), and then those with the highest percent recipient parent genome would be intermated, the progeny would be grown out, and the process would be repeated [42]. This process would be repeated for as many generations as are needed to recover the desired percent recipient parent genome; this should be possible in three or four generations. Systematic design would allow the simultaneous introgression of multiple QTL conferring SHGD, both in fewer generations than MABC and without the need for parallel pyramiding populations. 

Without access to a donor line with a single large-effect QTL, recurrent selection can be implemented to improve SHGD within a breeding program [19]. Molenaar et al. [19] evaluated the response of SHGD to recurrent selection by creating four populations where populations 1 and 2 shared a common parent with each other (denoted as C_0_P), and populations 3 and 4 shared a common parent with each other. The F_1_ plants forming populations 1 and 2 (denoted as C_0_-F_1_) were induced, and self-pollinations on all fertile D_0_ plants (using SHGD) were attempted. Five D_1_ ears with the highest seed set were selected (denoted as C_1_-P), and the progeny plants were crossed according to a half-diallel design (denoted as C_1_-F_1_) [19]. Individuals from each generation (C_0_-P, C_0_-F_1_, C_1_-P, and C_1_-F_1_) were then induced and phenotyped for SHGD [19]. Haploid plants of two entries from the C_1_-F_1_ generation were treated with colchicine, to serve as controls [19]. In contrast, populations 3 and 4 underwent four cycles of recurrent selection for SHGD, without using a diallel design [19]. D_1_ lines derived from ears with the highest seed set of the C_0_-F_1_ haploids were selected and recombined by pollinating the selected lines with a bulk of pollen from all of these lines, forming the C_1_-F_1_ generation [19]. Haploids were induced, and D_1_ plants with the highest seed set were again recombined, except this time with the exclusion of self-pollination, by using a pollen bulk of all other D_1_ lines, resulting in the C_2_-F_1_ generation. Haploids were induced, and the resulting D_1_ lines descending from D_0_ ears having greater than eight kernels were selected to form the C_3_-P generation [19]. These selected lines with high kernel set were pollinated with a bulk of pollen from all other C_3_-P lines, to exclude self-pollination. Haploids were derived from all generations of both populations, with the exception of C_1_-P and C_2_-P, and they were phenotyped for SHGD [19]. 

In both populations of the diallel experiment, the level of SHGD was significantly higher in the C_1_ generation than the C_0_, as were the additive variance components [19]. Furthermore, the heritability on an entry mean basis was over 0.90 [19]. In this experiment, using only recurrent selection, both populations underwent an increase in SHGD upon selection [19]. The increase of SHGD in population 4 was quadratic and was greatest between the C_2_ and C_3_ generations. The increase in population 3 was linear and occurred after each generation [19]. These results indicate that it is possible to select for SHGD, using recurrent selection, when SHGD is conferred by multiple loci [19]. However, more time would be required to use recurrent selection, and introgression of multiple SHGD loci into elite germplasm would be more costly compared to utilizing a single major locus. Nonetheless, regardless of the number of loci and method of introgression, SHGD would have a profound impact on the DH breeding process. 

## 4. Implications of Using SHGD in Maize Breeding

### 4.1. Utilization of SHGD within a Maize Breeding Program

There appear to be different mechanisms responsible for SHGD in maize, and their quantitative nature makes utilization difficult [19,33,34,38]. However, utilization of the SHGD systems from Ren et al. [23], or Ren et al. and Trampe et al. [21,22], would be much simpler because they are based on a single locus with large effect. Furthermore, the chromosome 5 locus identified by Ren et al. and Trampe et al. [21,22] displays additive gene action. The mode of gene action is still unknown for the QTL identified by Ren et al. [23], as segregation distortion mapping was used for its detection. 

Once incorporated in a breeding program, GS can be coupled with SHGD and performed on haploid plants, instead of DH lines in the following generation. Wu et al. [17] proposed that a minimum threshold for the haploid genome doubling rate of 20% is needed to implement GS economically at the haploid stage. While 80% of genotyped haploid plants are not capable of producing offspring, this approach would focus efforts for developing DH lines on the most promising genotypes based on marker data and reduce costs in the DH nursery. This threshold should be reasonable in most genetic backgrounds, given the additive gene action of the allele, large positive general combining ability, and a rate of SHGD of up to 46% in F_1_ plants derived from C_0_ inbreeds [20]. Furthermore, using SHGD should produce a similar, or even greater number of fertile haploids than colchicine [19]. Coupling SHGD with conducting GS using haploid individuals could drastically reduce the labor associated with DH line production, and subsequently the total costs, due to advancing a reduced number of individuals per population to the genome doubling stage. This however, assumes the costs associated with GS and SHGD are lower than the costs of labor and consumables associated with using a chemical doubling agent for genome doubling of haploid individuals. 

### 4.2. Advanced Applications of SHGD in Maize Breeding

SHGD could increase the efficiency of new applications implementing DH technology, such as using an HIL to induce genome editing mutations [43] and coupling genomic selection with genome editing to select favorable mutations by using promotion of alleles by genome editing (PAGE) [44]. Recently, Kelliher et al. [43] demonstrated that *Cas9* and guide RNA for delivering clustered regularly interspersed short palindromic repeats (CRISPR)-Cas9 induced mutations could be inserted by transformation into an HIL. It was then determined that the haploid induction process could induce targeted mutations in the resulting haploid progeny [43]. Because the HIL genome and the transgenic event associated with CRISPR-Cas9 are not transmitted to haploids during the haploid induction process, the result is non-transgenic edited plants [43]. A pitfall of CRISPR-Cas9 is that the efficiency of recovering plants with the desired edit can be quite low, with ~10% of the progeny carrying the target event, depending on many variables, including the target gene and guide RNA construction [45]. Therefore, very few haploid plants would likely carry the mutation event. It would, therefore, be important to maximize the doubling rate of D_0_ haploids, to maximize the likelihood of recovering a DH line possessing the target event. Therefore, if SHGD is present in the line being edited, and the targeted gene is not associated with the locus conferring SHGD, it would have the potential to increase the number of derived mutated DH lines. It may be more feasible to use embryo rescue and tissue culture practices for regenerating edited progeny due to high efficiency in genome doubling with these methods. However, small breeding companies, or breeding programs in developing countries, may not have the infrastructure in place to provide access to facilities capable of utilizing embryo rescue; in such cases, SHGD could be particularly useful. 

Furthermore, it is conceivable to imagine that PAGE could be implemented to increase the frequency of favorable alleles in DH lines and their preceding haploids, following HI-edit. Briefly, PAGE uses gene editing to target specific QTL that do not currently possess favorable alleles, transforming them to favorable alleles [44]. PAGE is particularly effective in increasing the response to selection when editing between 5 and 20 QTL with moderate effect [44]. The recovered edited individuals then undergo GS, to select individuals possessing the edited favorable alleles. When using PAGE, the response to selection increases with the number of targeted QTL, indicating that this approach is best suited for traits controlled by many small- to moderate-effect QTL [44]. Coupling PAGE with DH technology has the potential to improve the response to selection in a maize breeding program, particularly if coupled with HI-editing. If SHGD is present in the genetic background being edited, it would be conceivable to imagine that PAGE could be conducted with greater efficiency than when using colchicine, due to the increase in the DH line production of SHGD. 

Implementing SHGD in maize breeding has the potential to alter the traditional DH breeding pipeline and improve its efficiency by increasing the rate of genome doubling beyond that of traditional chemical doubling agents, further increasing the genetic gain per cycle in a breeding program. Furthermore, SHGD could greatly benefit developing countries that may not have access to chemical doubling agents, nor the equipment needed to implement such a pipeline, thus providing them feasible access to DH technology. Organic breeding programs can also exploit SHGD as a means to implement DH technology, given that it eliminates the need for chemicals that are not allowed to be used in organic systems. Therefore, SHGD can allow for the implementation of previously infeasible technologies to be conducted earlier in the breeding cycle, such as conducting GS on haploid individuals and possibly coupling it with CRISPR-Cas editing conferred by an HIL, to conduct PAGE. Moreover, the feasibility of such technologies would increase if the source germplasm of SHGD possesses a single large-effect QTL with additive gene action, such as that identified by Ren et al. and Trampe et al. [21,22], due to its ease of introgression and maintenance within a population.
